# Supplementary material for: The Evolution and Characterization of the RNA Interference Pathways in Lophotrochozoa
Source: Genome Biol Evol. 2024 May 7;16(5):evae098. doi: 10.1093/gbe/evae098 (PMC11114477; doi:10.1093/gbe/evae098)
Supplement: evae098_Supplementary_Data [file evae098_supplementary_data.zip › Supplementary_materials.zip!/selected_miRNAs/NoGe_selected_miRNAs/known_miRNA_families_pdf_files/NMRB01000823.1_Notospermus_geniculatus_strain_Ushimado_scaffold823,_whole_genome_shotgun_sequence_21770.pdf]

Provisional ID	:	NMRB01000823.1_Notospermus_geniculatus_strain_Ushimado_scaffold823,_whole_genome_shotgun_sequence_21770
Score total	:	2747.8
Score for star read(s)	:	3.9
Score for read counts	:	2738.9
Score for mfe	:	2
Score for randfold	:	0
Score for cons. seed	:	3
Total read count	:	5384
Mature read count	:	5273
Loop read count	:	0
Star read count	:	111

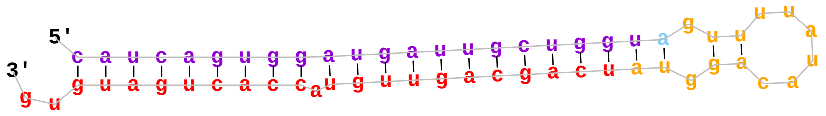

Genome coordinates: NMRB01000823.1 23582-23641

pre-miRNA sequence:
CAUCAGUGGAUGAUUGCUGGUCAUCAGUGGAUGAUUGCUGGUAGUUUUAUACAGGUAUCAGCAGUUGUACCACUGAUGUG

Mature sequence: UCAGCAGUUGUACCACUGAUGUG

Aligned mature sequence of the *N. geniculatus* pre-miRNA with the top 5 hits from the BLAST search against the pre-miRNA mirGeneDB set:

```
                            ******************** **
Nge_Mir-1992-P2             ucagcaguuguaccacugaugug
Cte-Mir-1992_3p             ucagcaguuguaccacugaugug
Obi-Mir-1992_3p             ucagcaguuguaccacugauuug
Ovu-Mir-1992_3p             ucagcaguuguaccacugauuug
Esc-Mir-1992_3p             ucagcaguuguaccacugauuug
Lgi-Mir-1992-P2_3p          ucagcaguuguaccacugauuug
```
